# Genetic and Epigenetic Regulation of Aortic Aneurysms

**DOI:** 10.1155/2017/7268521

**Published:** 2017-01-01

**Authors:** Ha Won Kim, Brian K. Stansfield

**Affiliations:** ^1^Vascular Biology Center, Augusta University, Augusta, GA, USA; ^2^Department of Pediatrics, Medical College of Georgia, Augusta University, Augusta, GA, USA

## Abstract

Aneurysms are characterized by structural deterioration of the vascular wall leading to progressive dilatation and, potentially, rupture of the aorta. While aortic aneurysms often remain clinically silent, the morbidity and mortality associated with aneurysm expansion and rupture are considerable. Over 13,000 deaths annually in the United States are attributable to aortic aneurysm rupture with less than 1 in 3 persons with aortic aneurysm rupture surviving to surgical intervention. Environmental and epidemiologic risk factors including smoking, male gender, hypertension, older age, dyslipidemia, atherosclerosis, and family history are highly associated with abdominal aortic aneurysms, while heritable genetic mutations are commonly associated with aneurysms of the thoracic aorta. Similar to other forms of cardiovascular disease, family history, genetic variation, and heritable mutations modify the risk of aortic aneurysm formation and provide mechanistic insight into the pathogenesis of human aortic aneurysms. This review will examine the relationship between heritable genetic and epigenetic influences on thoracic and abdominal aortic aneurysm formation and rupture.

## 1. Introduction

Aortic aneurysm formation is the result of a thinning medial layer and deterioration of the elastic lamina resulting in weakening of the tensile strength of the arterial wall. Aortic aneurysms are commonly identified in the thoracic and infrarenal aorta, with the latter referred to as abdominal aortic aneurysms (AAA). AAA represent the majority of aortic aneurysms and are classically associated with dyslipidemia, male sex, older age, smoking, and hypertension [1, 2]. The expansion of AAA is not a passive process but more closely mimics chronic inflammatory diseases characterized by hematopoietic cell infiltration and degradation of the extracellular matrix and vascular structures. Close association of inflammatory cells with breaks in the elastic lamina and the presence of reactive oxygen species suggests that AAA is an indolent process that eventually reaches a stress point resulting in aneurysm rupture [1, 3].

Thoracic aneurysms, on the other hand, are relatively rare and exhibit a strong heritable pattern. Approximately 20 percent of persons with thoracic aneurysms have a family history of aortic aneurysms. The relationship between thoracic aortic aneurysms and family history is strongest in first-degree relatives with 10-fold increased risk [4]. Syndromes associated with thoracic aortic aneurysms include Marfan syndrome (MFS), Loeys-Dietz syndrome (LDS), Ehlers-Danlos syndrome (EDS), familial thoracic aortic aneurysms and dissections (TAAD), autosomal dominant polycystic kidney disease (ADPKD), bicuspid aortic valve (BAV), and neurofibromatosis type 1 (NF1). Of these, MFS is the most common familial connective tissue disorder associated with thoracic aortic aneurysm, but each of these heritable syndromes shed light on the pathogenesis of aortic aneurysm formation [5]. A comprehensive understanding of heritable gene mutations and epigenetic modifications associated with aortic aneurysms will allow scientists and clinicians to design effective therapies and identify disease-specific biomarkers for tracking progression and risk for aneurysm rupture.

## 2. Syndromes Associated with Aortic Aneurysms

Marfan syndrome is the result of mutations in the* FBN1* gene on chromosome 15, which encodes fibrillin-1, an extracellular matrix (ECM) protein that forms microfibrils and controls vessel elasticity. Fibrillin-1 plays a vital role in maintaining the vascular architecture via transforming growth factor- (TGF-) *β* signaling, a cytokine that controls cell proliferation and differentiation [51, 52]. The importance of TGF-*β* signaling in maintaining vascular integrity was confirmed by the identification of mutations in the TGF-*β* receptor genes 1 and 2* (TGFBR1* and* TGFBR2)*, which cause Loeys-Dietz syndrome [53]. Similar to Marfan syndrome, mutations in both* TGFBR1* and* TGFBR2* result in disruption of collagen and elastin fiber biology in the vessel wall and aortic aneurysm formation [9]. Mutations in* TGFBR2* have also been linked to familial thoracic aortic aneurysms and dissections (TAAD), a syndrome associated with aneurysms of the ascending aorta and aortic dissections at relatively early stages of dilatation [54, 55]. TAAD represents a heterogeneous population of inherited disorders with mutations in myosin heavy chain-11* (MYH11)* and *α*-smooth muscle actin-2* (ACTA2)* among several, which are also linked to TAAD [15, 17, 19–21, 56–61].

Autosomal dominant polycystic kidney disease is commonly associated with intracerebral aneurysms but has been linked to thoracic aortic aneurysms and dissection as well [62, 63]. The etiology of this risk increase is thought to be multifactorial, as persons with ADPKD have increased prevalence of hypertension, a risk factor for thoracic aneurysms and AAA, and mutations in the* PKD1* and* PKD2* genes increase vascular smooth muscle cell (VSMC) apoptosis and induce dissecting aneurysms in mice [23, 63, 64]. While reports suggest that persons with APDPKD have an increased risk of AAA, a large cohort study failed to demonstrate an increased AAA prevalence in ADPKD [24]. Likewise, cardiovascular manifestations of neurofibromatosis type 1 occur in up to 10% of patients and tend to occur in adolescents and young adults [25]. Mutations in the* NF1* tumor suppressor gene increase VSMC proliferation and apoptosis, while mice with inactivating mutations in* Nf1* develop more frequent and severe aortic aneurysms than mice without the* Nf1* mutation [26, 27].

## 3. Candidate Genes Contributing to Aortic Aneurysm

The emergence of gene sequencing technology has greatly enabled the systematic search for candidate mutations and single nucleotide polymorphisms (SNPs) associated with aortic aneurysm formation. Much of this focus has been on identifying genes associated with AAA as the heritability of thoracic aneurysms is more commonly recognized [65], although mutational analysis of nonsyndromic thoracic aneurysms has gained interest. Recently, genome-wide association studies (GWAS) have identified several candidate SNPs related to nonsyndromic thoracic aneurysms and AAA [10, 66–68].  Table 1 summarizes genomic and epigenomic relationships with thoracic and abdominal aortic aneurysms. Many of these candidate genes require additional verification and must demonstrate functional plausibility in the pathogenesis of aortic aneurysms.

### 3.1. Extracellular Matrix Proteins

Fibrillin-1, coded by the* FBN1* gene, is an extracellular matrix glycoprotein that helps maintain the structural integrity of elastic fibers and other connective tissues. Mutations in the* FBN1* gene have been identified in 70–93% of persons who meet the diagnostic criteria for Marfan syndrome with more than 1,000 unique* FBN1* mutations observed in this population [6–8, 69]. GWAS studies of patients with sporadic thoracic aneurysms identified five independent SNPs in the* FBN1* coding region on chromosome 15, which may have predictive and diagnostic ability in nonsyndromic thoracic aortic aneurysms [7].

Fibulins (FBLN) are elastin-binding proteins that associate with fibronectin-containing fibers and lamina within the aorta and other large arteries [28]. FBLN5 functions as a bridging peptide to promote the adhesion of vascular endothelial cells. Mutations in the* FBLN5* gene result in cutis laxa and are potentially linked to age-related macular degeneration, a disease characterized by degradation of the elastin support structures [70]. Further,* Fbln5* knockout mice exhibit fragmentation of the elastic lamina and downregulation of FBLN5 is associated with aortic dilation in humans, which supports a role for FBLN5 in the pathogenesis of AAA [29, 71]. However, Badger et al. examined peripheral blood samples from 230 persons with AAA and 278 controls for three common SNPs in the* FBLN5* gene locus [30]. The frequency of each SNP was similar between cases and controls.

Degradation of collagen, a major component of the extracellular matrix (ECM), contributes to aneurysm formation and rupture due to a loss of tensile strength within the aorta. The* COL3A1* gene encodes a type III collagen found in the vascular wall that provides extensibility to the aorta. Mutations in the* COL3A1* gene cause Ehlers-Danlos Syndrome type IV [8] and hemizygous deletions have been linked to familial aortic aneurysm rupture [11]. Similar results were identified in* COL3A1* haploinsufficient mice [12, 13]. However, SNP analysis of AAA and control patients for the T581C variant of* COL3A1* failed to demonstrate a difference in the allele frequency between study populations [14].

Mutations in the* ELN* gene, encoding elastin (ELN), result in Williams Syndrome, a syndrome characterized by supravalvar aortic stenosis, cutis laxa, and other disorders of connective tissue [72]. Elastin is a highly elastic protein found in the lamina of muscular arteries and accommodates arterial dilation and facilitates recoil.* ELN* knockout mice develop severe hypertension and die shortly after birth, while* ELN* heterozygous mice develop muscularized arteries and moderate hypertension [73]. Additionally, decreased elastin expression potentiates VSMC proliferation and increases arterial fragility. Thus, elastin appears to play an important role in maintaining vascular wall integrity under stress. However, a propensity for aortic aneurysm formation has not been demonstrated in* ELN* mutant mice. SNPs in the* ELN* gene have been identified in patients with a strong family history of AAA, but cohort studies of nonrelated AAA and control patients have failed to show a difference in SNP frequency [14].

Matrix metalloproteinases (MMP) are zinc-containing peptidases that are biologically active molecules involved in the degradation of ECM proteins and play an important role in cell proliferation, migration, and apoptosis. Matrix metalloproteinase-2 (MMP2) is highly expressed in VSMC and AAA in both mice and humans, but mutational analysis of AAA and control patients has failed to reveal a predictive SNP in the* MMP2* gene [31, 74–79]. Similarly, MMP9 and MMP12 appear to participate in the pathogenesis of AAA in mouse models, but no association between* MMP9* or* MMP12* gene variants and AAA has been identified [32, 33, 77, 80]. MMP3, on the other hand, is highly expressed in human AAA and a common SNP in the promoter region of the* MMP3 *gene was found to correlate positively with AAA formation and coronary aneurysms in humans [34, 35]. Tissue inhibitor of metalloproteinases (TIMPs) inhibits MMP activation and plays a pivotal role in determining the influence of the ECM and cell adhesion molecules on VSMC function within the vascular wall. SNP analysis of the* TIMP1* gene has yielded conflicting data with one study showing an association with AAA patients without a family history of AAA and a larger meta-analysis failing to show an association between* TIMP1* polymorphisms and AAA [14, 81]. No association between AAA and* TIMP2* or* TIMP3* has been identified [14].

### 3.2. TGF-*β* Pathway

Transforming growth factor-*β* (TGF-*β*) is a cytokine that regulates a variety of cellular functions, including differentiation, transformation, and proliferation. Dysregulated TGF-*β* signaling has been implicated in the pathogenesis of both thoracic aneurysms and AAA. The TGF-*β* superfamily members can bind to fibrillin; therefore, fibrillin-1 deficiency impairs matrix sequestration of latent TGF-*β*, leading to uncontrolled secretion of TGF-*β* and upregulated TGF-*β* signaling [82, 83]. More recently, noncanonical TGF-*β* signaling through SMAD proteins has been shown to induce thoracic aortic aneurysm formation via the mitogen-activated protein kinase (MAPK) pathway [84–86]. These later observations are particularly interesting as SMAD mediated MAPK activation overlaps with other syndromes with a predisposition for thoracic aneurysm formation.

Likewise, TGF-*β* signaling has been implicated in the inflammatory AAA animal models and human AAA. However, TGF-*β* appears to play a protective role in AAA. TGF-*β* and TGFBR2 are poorly expressed in human AAA tissue samples compared to controls and disruption of TGF-*β* signaling prevents AAA formation in the elastase and angiotensin (AngII) animal models of AAA [87–90]. Interestingly, AngII increases the expression and activation of all TGF-*β* isoforms and TGFBR1 and TGFBR2 within both the thoracic and abdominal murine aorta [91–93]. Analysis of the* TGFB1* and* TGFBR1 *genes failed to reveal an association with human AAA [14, 81], but two SNPs in* TGFBR2* (rs1036095 and rs4522809) were associated with AAA [10]. Additionally, mutations in the* SMAD3* gene have been linked with aneurysm-osteoarthritis syndrome, which further demonstrates the critical role of noncanonical TGF-*β* in aortic aneurysm formation [22].

### 3.3. Smooth Muscle-Related Proteins

Smooth muscle cells, along with the elastic lamina and ECM, provide the structural integrity of the vascular wall. Thus, mutations affecting VSMC function and/or their ability to maintain the ECM could render the aorta vulnerable to dilation and/or rupture. Mutations in smooth muscle myosin* (MYH11)*, coding for smooth muscle myosin heavy chain, have been linked to isolated familial thoracic aortic aneurysm [16]. Ascending aortic aneurysms in patients with* MYH11* mutations are accompanied by a high rate of patent ductus arteriosus (PDA), but disruption of this locus is responsible for only a small fraction of sporadic PDA [16, 94]. Similarly, mutations in *α*-smooth muscle actin* (ACTA2)* are associated with several manifestations of cardiovascular disease including thoracic and cerebral aneurysms, myocardial infarction, and neurovascular malformations [18]. Given that ACTA2 is a component of smooth muscle cells and also a transcriptional target of TGF-*β* signaling, mutations in this gene likely affect VSMC functions and impair vascular contraction.

### 3.4. Lipid Metabolism

Apolipoprotein E (APOE) binds to the low-density lipoprotein receptor (LDLR) to clear lipoprotein particles from the blood and is critical for lipid and lipoprotein metabolism. Mutations in the* LDLR* gene are linked to familial hypercholesterolemia and type III hyperlipidemia, which significantly increase the susceptibility to premature and severe atherosclerosis [95]. GWAS have identified mutations in the* LDLR*, located on chromosome 19p13.2, and the low-density lipoprotein receptor-related protein 1 (LRP1), located on chromosome 12q13.3, which are associated with genetic susceptibility to AAA [36, 37, 96]. Additionally, several* APOE* polymorphisms modify the risk for atherosclerosis and AAA. Multiple studies have examined APOE*∗*2, -*∗*3, and *∗*4 alleles in patients with AAAs, and individuals with the E3/E4 genotype showed a markedly lower AAA expansion rate than those with the E3/E3 genotype [38]. Interestingly the E4 genotype is associated with shorter lifespan in several cross-sectional epidemiologic studies [97–99].

### 3.5. RAS-Related Pathway

Neurofibromin is encoded by the* NF1* tumor suppressor gene and functions as a negative regulator of p21^Ras^ (Ras) activity in circulating hematopoietic and vascular wall cells [26, 100–103]. Mutations in the* NF1* gene are associated with neurofibromatosis type I and Watson syndrome, a variant of NF1 [104]. The incidence of cardiovascular disease and aneurysm formation in persons with NF1 approaches 10% [105–109]. Most aortic aneurysms in NF1 are located in the thoracic descending aorta, but animal models of* Nf1* mutant animals demonstrate a strong predisposition for infra-renal AAA as well [27]. Broader epidemiologic studies of NF1 patients and aneurysm risk have yet to be performed.

PTPN11 (SH2 domain-containing protein tyrosine phosphatase-2) is encoded by the* PTPN11* gene and regulates a variety of cell functions, including mitogenic activation and gene transcription. Mutations in the* PTPN11* gene cause Noonan syndrome, a relatively rare inherited disorder characterized by accelerated Ras-MAPK signaling transduction. Persons with Noonan syndrome are at increased risk for dilation of the aortic root and aneurysm formation in the ascending aorta [39–41]. It is mechanistically plausible that other inherited mutations in the Ras pathway may modify the risk of thoracic aneurysm formation, but animal studies and/or human data supporting this hypothesis are limited.

## 4. Epigenetic Risk Factors

Epigenetics refers to heritable and acquired modifications to the genome that alter gene expression without changing the DNA sequence. In some instances, epigenetic modifications are stable and passed on to future generations, but many modifications are relatively dynamic and responsive to environmental cues. Epigenetic modifications include DNA methylation, histone modifications, and noncoding RNA, which can directly interact with the primary nucleotide sequence and regulate gene expression. Methyltransferases are enzymes that methylate DNA and the supporting elements including histones to alter gene activity and chromatin structure. DNA methylation occurs naturally as a result of aging and cell differentiation but is also recognized as an important modifier of disease risk.

Similarly, posttranslational histone modification by the addition or removal of methyl or acetyl groups, phosphorylation, ubiquitylation, and sumoylation results in suppression or activation of gene transcription by altering the chromatin structure or recruiting additional histone modifiers. Modifications that increase chromatin condensation restrict access of transcription factors to the gene target, while modifications that decrease chromatin condensation lead to a more open chromatin structure and increase gene expression. Histone modifying enzymes, such as histone deacetylases (HDACs), histone methyltransferases (HMT), and histone acetyltransferases (HAT), have been implicated in cardiovascular disease, cancer initiation and propagation, and Alzheimer's disease. Presently, epigenetic effects on the frequency, severity, and progression of thoracic aneurysms and AAA are limited with most of the data on AAA being inferred from studies of atherosclerosis and other inflammatory conditions. However, atherosclerosis and AAA are distinct clinical entities with overlapping risk factors and disease mechanisms. Thus, interpretation of study results from related cardiovascular diseases must be viewed with some caution.

### 4.1. Epigenetic Modification in Matrix Degradation

MMP expression and activation are a hallmark feature of AAA. In particular, MMP-2 and MMP-9 are highly expressed in human and murine AAA and likely have some role in thoracic aortic aneurysms [75, 77, 110–112]. Increased expression of active MMPs predicts severity of other chronic inflammatory diseases such as rheumatoid arthritis, chronic kidney disease, and degenerative inflammatory disorder [113–115]. Demethylation of the* MMP2* gene promoter increases MMP-2 expression in noninvasive breast cancer cell lines and cells treated with trichostatin A, an HDAC inhibitor, demonstrated increased histone acetylation and reduced* MMP2* mRNA expression [116, 117]. Likewise, methylation and acetylation of* MMP9* result in downregulation of MMP-9 expression and inhibit MMP-9 binding to the CREB transcription factor via Class II major histocompatibility complex transactivator, respectively [118, 119]. Interestingly, HDAC2 binding to the* MMP9* promoter region suppresses MMP-9 expression and suggests that epigenetic modification of MMPs is tightly regulated [120]. While MMP expression is highly associated with human and murine aortic aneurysms, the regulation of MMP expression in cardiovascular tissues or primary cells remains poorly understood.

Classes I and II HDACs contain a conserved HDAC domain but differ in their expression patterns with broad expression of Class I HDACs and more limited expression of Class II HDACs. In human AAA, Galán et al. recently reported increased expression of HDACs 1, 2 (Class I), 4, and 7 (Class II) in human AAA compared to aortic tissue samples from healthy organ donors [121]. Further, the use of HDAC inhibitors significantly reduced AngII-induced AAA in* APOE* knockout mice via downregulation of MMP-2 and MMP-9 in the vascular wall [122]. Administration of Class I or Class II HDAC inhibitors were efficacious in reducing the frequency and severity of AAA in mice, which further supports the hypothesis that epigenetic modifications are critical to the pathogenesis of aortic aneurysms and HDAC inhibitors represent a promising therapeutic strategy [121].

Epidemiologic data suggests that carriers of the* MTHFR* C677T allele are at increased risk for AAA [42–44]. Persons with this polymorphism express a heat-labile MTHFR enzyme with reduced activity and increased plasma homocysteine levels, which is a known risk factor for AAA and growth rate [123–125]. Homocysteine is considered a potent inhibitor of methyltransferases by increasing the intracellular accumulation of s-adenosyl homocysteine and sequestering methyl donor groups [126, 127]. In support of this hypothesis,* MTHFR* knockout mice exhibit global DNA hypomethylation and enhanced atherosclerosis, although an increased susceptibility to AAA in animal models has not been demonstrated [128]. The role of methyltransferases in AAA is an emerging area of interest.

### 4.2. Epigenetic Modification in Smoking

Smoking is a strong risk factor for AAA; however, the underlying mechanisms for this risk association are poorly understood. Exposure to prolonged smoking alters the epigenome, particularly in high-turnover cell lines such as bone marrow-derived leukocytes. Current smoking status and prenatal exposure to cigarette smoke strongly influence the methylation signature of DNA [129–132]. In one of the most insightful studies of cigarette exposure and the epigenome, Stephanie London and colleagues conducted a meta-analysis of 15,907 individuals to identify the methylation footprint of smokers and nonsmokers across the epigenome [133]. They showed that 1/3 (>7,000) of currently recognized human genes have smoking-associated methylation patterns. Interestingly, CpGs (cytosine-rich methylation sites) that are causally related to cardiovascular diseases showed the strongest correlations for both current and former smokers as compared to persons who have never smoked [133]. Former smokers appear to have a methylation signature that more closely mimics nonsmokers; however, smoking cessation does not appear to fully reverse these changes [133–135].

Examination of specific gene promoters has identified similar changes in DNA methylation for candidate genes associated with AAA formation including protease-activated receptor-4 (PAR4 or F2RL3) and 15-lipoxygenase (ALOX15). Breitling et al. examined the methylation status of 27,000 CpGs to show that methylation of the* F2RL3 *gene was reduced by 12% in smokers when compared with nonsmokers [130]. Further, CpG methylation correlated negatively with the quantity of cigarettes smoked and positively with duration of smoking abstinence. Broader examination of intergenic CpG methylation has identified similar relationships between smoking and altered DNA methylation [136, 137]. Methylation status of the* Alox15* promoter directly affects gene expression with heavy smokers showing a substantial increase in 15-lipoxygenase levels compared to nonsmokers [138, 139]. 15-Lipoxygenase has been implicated in the pathogenesis of AAA and pharmacologic or genetic inhibition of 5-lipoxygenase signaling has demonstrated efficacy in preventing AAA in animal models [45, 46]. However, further epigenomic studies of human AAA are needed to demonstrate the precise mechanisms relating smoking to AAA formation.

### 4.3. Epigenetic Modification in Aging

Older age is associated with higher risk for AAA and aging-related epigenetic changes have been proposed as a potential mechanism for this increased risk. After methylation patterns have been established during human development, progressive, time-dependent, global hypomethylation occurs [140–142]. Most of these observations have been made in primary cell lines or animal models of cancer and other age-related diseases with few observations in older adults with AAA. Studies of atheroma in humans and atherogenic mice exhibit a global loss of genomic 5-methyl-Cytosine content, a common occurrence in neoplastic cells [143–145]. 5-Methyl-C is produced by transfer of a methyl group from S-adenosylmethionine, a process that is inhibited by decreased MTHFR activity and elevated homocysteine levels associated with aging [146–148]. One investigator proposes that DNA methylation measures the cumulative effect of the epigenetic maintenance system and may predict many age-related diseases [149]. These observations are likely relevant to aging and increased risk for AA formation.

### 4.4. Epigenetic Modification in Inflammatory Responses

Chronic inflammation characterizes AAA, wherein inflammatory leukocytes and cytokines as well as reactive oxygen species are found within the vascular wall in human AAA and animal models of AAA. Inflammation also influences the epigenome of circulating leukocytes and vascular wall cells. Ryer et al. examined the genome-wide DNA methylation profiles of mononuclear cells isolated from 20 humans with AAA and 21 controls. Four genes with differential CpG methylation were identified: kelch-like family member 35 (*KLHL35*), calponin 2 (*CNN2*), serpin peptidase inhibitor clade B (ovalbumin) member 9 (*SERPINB9*), and adenylate cyclase 10 pseudogene 1* (ADCY10P1) *[47]. For three of these genes (*CNN2*,* SERPINB9*, and* ADCY10P1*), methylation more closely correlated with the presence of AAA than with either age or smoking status and suggests that the methylation status of these genes may represent an independent and additive risk factor for AAA.

Changes in DNA methylation within the promoter region of immunomodulatory cytokines may also affect risk for AAA. Treatment of cultured human lymphocytes with interleukin-6 increases global methylation in these cells [114]. Promoter methylation regulates the expression and activity of other inflammatory cytokines implicated in AAA formation including TNF-*α*, IL-1*β*, and monocyte chemotactic protein-1 (MCP-1 or CCL2) [150–154]. Likewise, reactive oxygen species (ROS) can increase histone acetylation via modulation of HAT activity to promote inflammation in several cell lines [155–157]. Hydrogen peroxide induces gene acetylation by increasing HAT activity and suppressing HDAC activity [158]. Thus, blunting oxidative stress may be a promising strategy to suppress inflammatory conditions such as AAA.

### 4.5. Noncoding RNAs in AAA

Noncoding RNAs (ncRNA) are functional RNA transcribed from DNA but fail to be translated into proteins. ncRNA, including transfer RNA (tRNA), ribosomal RNA (rRNA), microRNA (miRNA), and long noncoding RNA (lncRNA), are abundant, biologically active molecules that modify gene expression. MicroRNA are short (21–25 nucleotides), single-stranded RNA molecules that mimic small interfering RNA and function in RNA silencing and posttranscriptional regulation of gene expression.

A significant number of miRNAs are differentially expressed in AAA and normal aortic tissue samples; however, most studies fail to identify similar miRNA trends, which is likely due to the heterogeneity of AAA tissue [159]. Pahl et al. showed that AAA have increased expression of miR-181a, -146a, and -21 and lower expression of miR-133b, -331-3p, -133a, -30c, and -204 when compared with control biopsies [160]. Other miRNA with differential expression in AAA and control tissue include miR-126, -20a, -27a, -155 -221, -222, -223, -124a, -29b, and let-7 [161]. Of these, miR-21 and -146a expressions are consistently elevated in AAA compared to healthy aorta, and miR-21 has been shown to inhibit VSMC apoptosis and protect against AAA formation in* APOE* knockout mice [48]. Several other miRNA, including miR-146a, promote VSMC survival and participate in the pathogenesis of AAA [49, 162, 163]. Interestingly, miR-29b has been implicated in both AAA and thoracic aneurysms associated with Marfan syndrome [49, 50].

Analysis of circulating miRNAs in peripheral blood has also yielded useful insight into AAA. Zhang et al. identified increased expression of miR-191-3p, -455-3p, and -1281 in the whole blood of AAA patients compared to controls, while a separate study showed decreased expression of miR-126, -124a, -146a, -155, -223, -29b, -15a, and -15b in AAA [164, 165]. Stather et al. reported reduced expression of let-7e, miR-15a, and -196b in the peripheral blood of AAA patients and increased expression of miR-411 as compared to controls [165]. The increased expression of some miRNA in AAA tissue and decreased expression in peripheral blood (i.e., miR-146a and miR-29b) demonstrate the complex nature of using miRNA as biomarkers of disease progression. Larger studies comparing miRNA expression in peripheral blood and primary tissue would shed considerable light on the role of miRNA in AAA formation.

Recently, an intense interest in the role of noncoding RNAs has broadened our understanding of the influence of the epigenome on aortic aneurysm formation. After showing that miR-29b is significantly downregulated in two animal models of AAA, Maegdefessel et al. showed that administration of an anti-miR to further diminish miR-29b expression in the aortic wall reduced aorta dilation and AAA formation [49]. Conversely, administration of a pre-miR-29b to increase miR-29b expression within the aorta exacerbated AAA formation and aneurysm rupture (63% versus 33%). In both animal models and human AAA tissue samples, miR-29b appears to target genes that encode extracellular matrix proteins including Col1a1, Col3a1, Col5a1, and ELN as well as matrix metalloproteinases (i.e., MMP9). This same group identified a role for miR-29b in thoracic aortic aneurysms in a murine model of Marfan syndrome. Contrary to AAA, aortas from* Fbn1* mutant mice (Marfan) expressed higher quantities of miR-29b, which corresponded with enhanced apoptosis, increased MMP expression, and suppression of nuclear factor kappa-light-chain-enhancer of activated B cells (NF-*κ*B) activity, a repressor of miR-29b [50]. Boon et al. examined the aortas of young and old mice as well as tissue samples of human thoracic aneurysms to identify patterns of micro-RNA expression. The miR-29 family was highly expressed in aortas from older mice and was associated with downregulation of ECM protein expression [166]. Similar to the mice, human tissue samples of thoracic aortic aneurysm expressed high levels of miR-29b when compared to human controls.

While the data for a pathological role for miR-29b in AAA is compelling, other miRNAs have been implicated in AAA as well. miR-21 controls VSMC proliferation and apoptosis during AAA formation and upregulation of miR-21 has been demonstrated in AAA biopsy samples [48]. Nicotine enhances both miR-21 expression and AAA size in mice and AAA biopsy samples isolated from frequent smokers showed a twofold increase in miR-21 expression when compared with AAA from nonsmokers [48]. In both mice and humans, the increase in miR-21 expression was associated with reduced expression of phosphatase and tensin homolog (PTEN) and increased activation of protein kinase B (Akt).

Murine AAA exhibited increased expression of miR-24 with genetic deletion of miR-24 further enhancing murine AAA size and severity [167]. Chitinase 3-like 1 gene (Chi311), which promotes cytokine synthesis in leukocytes and VSMC migration, appears to be a potential target of miR-24 [167]. Additionally, miR-712 and its human homolog miR-205 suppress metalloprotease inhibitor activity in response to AngII to stimulate MMP activity in aortic VSMC and facilitate AAA formation [168]. Although miRNA influence aortic aneurysm formation and severity, it remains to be seen if targeting specific miRNA is a viable therapeutic approach for the prevention or treatment of human aortic aneurysms.

## 5. Conclusions

In this review, we provide a comprehensive assessment of the genetic and epigenetic landscape of aortic aneurysms. We have long appreciated the influence of inherited syndromes on aortic aneurysm formation and aneurysm rupture and the multiple environmental and familial risk factors associated with AAA, but more and more, we are gaining insight into the complexities of gene regulation and protein function in the pathogenesis of aortic aneurysms. Of these recent advances, miRNAs and epigenetic modifiers are particularly intriguing and may represent viable therapeutic targets for patients with aortic aneurysms as seen in other patient populations. Future work must include comparisons between findings in human tissue and animal models of aortic aneurysm to draw clinically relevant conclusions about the role of miRNAs or other epigenome modifiers in aortic aneurysm formation.

## Figures and Tables

**Table 1:** Summary of genetic and epigenetic relationships with TAAD and AAA.

Mutation	Syndrome	TAAD	AAA
FBN1 [5–8]	Marfan	+	
TGFBR1 [9], TGFBR2 [9, 10]	Loeys-Dietz	+	
COL3A1 [11–14]	Ehlers-Danlos	+	+/−
TGFBR2, MYH11 [15, 16], ACTA2 [17, 18] LOX [19], MAT2A [20], SMAD3 [21, 22]	Familial TAAD	+	
PKD1 [23, 24], PKD2 [24]	ADPKD	+	+/−
NF1 [25–27]	NF1	+	+/−
FBLN5 [28–30]		+	
ELN [14]	Williams		+
MMP2 [31], MMP9 [32], MMP12 [33]			+/−
MMP3 [34, 35]			+
TIMP1 [14]			+
LDLR1 [36, 37]			+
APOE E3/E3 [38]			+
PTPN11 [39–41]	Noonan	+	
MTHFR C677T [42–44]			+
ALOX15 [45, 46]			+
CNN2 [47]			+
SERPINB9 [47]			+
ADCY10P1 [47]			+
miR-21 [48], miR-146a [48]			+
miR-29b [49, 50]		+	+
